# Near-Complete Genome Sequence of Feline Immunodeficiency Virus from Colombia

**DOI:** 10.1128/MRA.00754-20

**Published:** 2020-08-13

**Authors:** Sueli A. Taniwaki, Tatiana Jiménez-Villegas, Nelson F. Santana-Clavijo, Taís F. Cruz, Sheila O. S. Silva, Juan D. Valencia-Bacca, Felipe Loaiza-Pedreros, Leonardo J. Richtzenhain, Fernando Ferreira, João P. Araújo Júnior, Paulo E. Brandão

**Affiliations:** a Department of Preventive Veterinary Medicine and Animal Health, School of Veterinary Medicine, University of São Paulo, São Paulo, São Paulo, Brazil; b Department of Microbiology and Immunology, Institute of Bioscience, São Paulo State University, Botucatu, São Paulo, Brazil; c Institute for Biotechnology, São Paulo State University, Botucatu, São Paulo, Brazil; d La Casa del Gato Feliz Veterinary Clinic, Bogota, Cundinamarca, Colombia; KU Leuven

## Abstract

Feline immunodeficiency virus (FIV) is an important pathogen of domestic and wild felids. Although serological tests suggest the presence of FIV in cats from Colombia, no molecular characterization has been reported. Here, we describe the near-complete genome of FIV subtype A from a Colombian domestic cat.

## ANNOUNCEMENT

Feline immunodeficiency virus (FIV) causes AIDS in cats, similar to human immunodeficiency virus (HIV) infection. Both retroviruses belong to the genus *Lentivirus*, and because of their similar genomic organization and pathology, FIV infections have been used as an experimental model for HIV vaccine development and investigation of virus-host interactions (1, 2). FIV strains are classified into six subtypes (A to F), based on *gag* and *env* genes; subtypes A and B are the most prevalent, with worldwide distribution (3). In Colombia, FIV prevalence ranges from 6.7 to 13%, based on serological tests (4, 5), and no data about molecular characterization exist.

Total DNA was extracted from whole blood from a naturally infected male Ragdoll cat from Bogota, Colombia, in 2018, using the DNeasy blood and tissue kit (Qiagen). The near-complete genome was amplified with Platinum SuperFi DNA polymerase (Invitrogen), using primers targeting the primer binding site and the repetition region of the 3′ long terminal repeat (6) of FIV proviral DNA (FIVpbs_For, 5′-CAG TTG GCG CCC GAA CAG GGA-C-3′ [positions 354 to 375]; FIVR2_Rev, 5′-GTG GGA GCC TCA AGG GAG AAC TC-3′ [positions 9381 to 9359, based on the Petaluma reference strain with GenBank accession number M25381]). Purified amplicons were subjected to library construction using the Nextera XT Index and Nextera XT DNA kits (Illumina). The library was sequenced using the MiSeq reagent kit v.3 (600 cycles) on a MiSeq system (Illumina), generating 1,523,068 reads. Reads were trimmed (quality limit, 0.01; minimum read length, 150 nucleotides [nt]) and *de novo* assembled using default parameters (minimum contig length, 5,000 nt) with CLC Genomics Workbench v.12.0.3 (Qiagen). *De novo* assembly of 913,185 reads after trimming (*Q*, >30) generated a contig with 9,056 nt, using 911,799 reads with 24,200-fold average coverage. The annotation of structural and accessory proteins was made based on the FIV Petaluma strain (subtype A). The primer sequences were removed and phylogenetic analyses were conducted with MEGA X (7) and BioEdit Sequence Alignment Editor (8) software.

**FIG 1:**
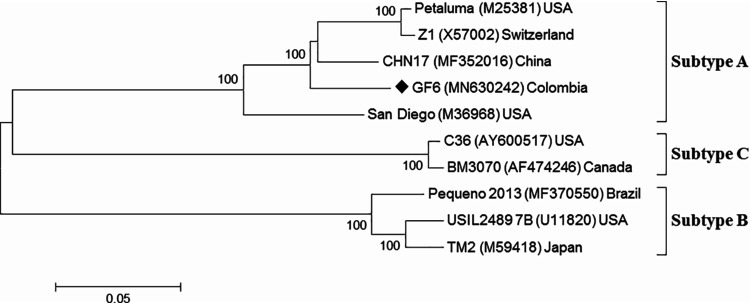
Phylogenetic tree of FIV near-complete genome sequences, constructed with the maximum likelihood method and the general time-reversible model. The diamond marks the sequence obtained in our study. Bootstraps of 1,000 replicates with values of >70 are shown at the nodes. The numbers in parentheses are GenBank accession numbers. The bar represents the substitution number per site.

The near-complete FIV genome sequence of 8,977 nt includes the entire coding regions of structural protein genes *gag* (nt 253 to 1605), *pol* (nt 1494 to 4868), and *env* (nt 5891 to 8461) and accessory protein genes *vif* (nt 4861 to 5616), ORF-A (nt 5617 to 5853), *rev* (nt 5891 to 6136 and 8581 to 8790), and ORF-D (nt 6337 to 6537), as well as the 3′ untranslated region (nt 8740 to 8954). Alignment and phylogenetic analysis of entire genome sequences demonstrated that FIV isolate GF6 from a Colombian cat belongs to subtype A (Fig. 1). Compared to the Petaluma strain, the full genome of GF6 showed nucleotide identity of 93.7%, whereas amino acid identities of 95.5, 96.0, 89.6, 92.4, 74.3, 90.1, and 83.8% were found for Gag, Pol, Env, Vif, ORF-A, Rev, and ORF-D proteins, respectively.

In South America, studies from Brazil and Argentina demonstrated circulation of subtypes B and E, using partial or complete genome sequences (9–14). Subtype A and an unknown subtype were also described in Brazil (15, 16), but no sequences are available in GenBank. The description of the complete coding sequence plus the 3′ untranslated region of FIV subtype A presented here improves the knowledge of the molecular epidemiology of FIV in Colombia and South America.

### Data availability.

The near-complete genome of FIV isolate GF6 from Colombia is available in GenBank (accession number MN630242), and the raw data are available in the SRA database (accession number PRJNA645463).
